# Digging Up the Human Genome: Current Progress in Deciphering Adverse Drug Reactions

**DOI:** 10.1155/2014/824343

**Published:** 2014-03-10

**Authors:** Shih-Chi Su, Wen-Hung Chung, Shuen-Iu Hung

**Affiliations:** ^1^Department of Dermatology, Drug Hypersensitivity Clinical and Research Center, Chang Gung Memorial Hospital, 199 Tung-Hwa North Road, Taipei 105, Taiwan; ^2^College of Medicine, Chang Gung University, 259 Wen-Hua First Road, Taoyuan 333, Taiwan; ^3^Institute of Pharmacology, School of Medicine, Infection and Immunity Research Center, VYM Genome Research Center, National Yang-Ming University, No. 155, Section 2, Linong Street, Taipei 112, Taiwan

## Abstract

Adverse drug reactions (ADRs) are a major clinical problem. In addition to their clinical impact on human health, there is an enormous cost associated with ADRs in health care and pharmaceutical industry. Increasing studies revealed that genetic variants can determine the susceptibility of individuals to ADRs. The development of modern genomic technologies has led to a tremendous advancement of improving the drug safety and efficacy and minimizing the ADRs. This review will discuss the pharmacogenomic techniques used to unveil the determinants of ADRs and summarize the current progresses concerning the identification of biomarkers for ADRs, with a focus on genetic variants for genes encoding drug-metabolizing enzymes, drug-transporter proteins, and human leukocyte antigen (HLA). The knowledge gained from these cutting-edge findings will form the basis for better prediction and management for ADRs, ultimately making the medicine personalized.

## 1. Introduction

Adverse drug reactions (ADRs) are side effects occurring within the approved dosage and labeling recommendations. Severe ADRs, which require hospitalization, are a significant clinical problem in drug therapy because they can be permanently disabling or result in death. The incidence of severe ADRs has been estimated at 6.2–6.7% in hospitalized patients and the incidence of fatal ADRs is estimated to be 0.15–0.3% [1]. From a clinical aspect, ADRs can be broadly divided into two types, type A and type B [2]. Type A reactions are considered as a magnification of a drug's therapeutic effect and represent the majority of ADRs. This type of condition is predictable from the known pharmacology of a drug and typically dose dependent. By contract, type B reactions are less common and do not involve the pharmacological effects of a drug. Moreover, most individuals are not susceptible to type B ADRs, which are, thus, being termed “idiosyncratic.” With the advance of the current understanding in their underlying mechanisms, some type B reactions now become potentially avoidable although totally unpredictable in the past.

In addition to the impact on health care, ADR remains a huge cost burden for pharmaceutical industry. It has been reported that 56 out of 548 newly approved drugs in the US either had to be withdrawn from the market or achieved a black box warning due to adverse reactions that were unpredicted by clinical trials from 1975 to 1999 [3]. Although controversial, there is an estimate that the cost of bringing a single new drug to market is US$802 million [4]. Thus, severe ADRs pose tremendous challenges both to patient care and to pharmaceutical development. The current successes in discovering specific genotypes that are highly associated with certain ADRs are encouraging; however, a more comprehensive understanding is essential for dealing with this complex problem. In this review, we discuss the pharmacogenomic techniques used to explore the pathogenesis of ADRs and summarize the current progresses concerning genetic associations and predictors for the occurrence of ADRs, with a focus on genetic variants for genes encoding drug-metabolizing enzymes, drug-transporter proteins, and human leukocyte antigen (HLA).

## 2. Pharmacogenomic Strategies for Studying ADR 

In the past few decades, many genes which are implicated in simple, monogenic disorders have been discovered by using linkage analysis and positional cloning approaches. However, these methods were less successful in mapping genes that are involved in complex diseases, like ADRs, because such diseases typically are caused by several genes, each with a portion of overall contribution. Researchers, thus, began to conduct the association studies using the candidate-gene approach to search for the statistical correlation between genetic variants and a disease. These genetic association studies, through which the relation of selected genes/genotypes with the etiological role of a disease in a group of population-based samples from affected and unaffected (case versus control) individuals was analyzed, are likely to be more useful than linkage studies for studying complex traits because they can have greater statistical power to find numerous genes of small effect [5]. In spite of its advantage, it has been reported that the association studies of the same disease using such candidate-gene approach are often inconsistent in their findings and that the first study to report an association often presents a stronger effect than that observed in subsequent studies [6].

With the completion of the human genome project [7] and the availability of comprehensive data on variability in human genome from the HapMap [8], huge strides have been made in our understanding of single nucleotide polymorphism (SNP) and the impact of interindividual genetic variants on the risk of complex diseases. These findings together with the development of modern methods and techniques allowing the prosecution of large-scale association studies have evolved the studies of complex disorders from the candidate-gene approach to the genomewide association study (GWAS). Unlike the candidate-gene approach that highlighted the selected genes, GWAS aims to analyze the genotype of SNPs throughout the whole genome, not simply focusing on those that are obvious candidates for effects on the disease of interest. Due to this open nature, GWAS does not require an initial hypothesis for exploring the genetic predisposing factors to a complex disease. However, a limitation of GWAS is that a large sample size is required to discover the SNPs with relatively low odds ratios [9]. This often harnesses the studies on severe, idiosyncratic ADRs which occur at very low frequencies, unless samples are collected via international collaboration [10].

In addition, advances in DNA sequencing technologies that allow substantial increases in sequencing content while dramatically decreasing the cost per base have facilitated the advent of high-throughput sequencing methods, often referred to as next-generation sequencing (NGS). These techniques, including whole-genome sequencing that reads the complete sequence of an individual's genome at a single time and whole-exome sequencing that captures only the parts of the DNA which code for proteins, have been successfully applied to numerous disease-targeted tests in disease diagnostics [11]. The central advantage of NGS over GWAS on exploration of genetic etiology of polygenic diseases is that NGS can directly identify the causal variants whereas GWAS primarily is designed for seeking markers that are intended to represent causal variation indirectly. Furthermore, these sequencing-based methods possess higher explorative power than does GWAS, enabling to discover the causal variations with low allele frequencies (<5%) in complex traits [12], although the development of chip-based genotyping advances greatly. Yet, until recently, studies of uncovering the genetic susceptibility to complex diseases or severe ADRs using NGS techniques are still very limited.

## 3. Drug-Metabolizing Enzymes

Interindividual differences in drug disposition have been recognized as important and common causes of adverse drug reactions [13]. Drug metabolism is generally classified into two phases, termed phase I and phase II. Phase I reactions encompass oxidation or reduction reactions, usually through the actions of cytochrome p450 oxidative enzymes or reductases. These phase I drug-metabolizing enzymes (DME) process the parent drugs for phase II reactions by creating a conjugation site on the drug. Subsequently, phase II DME acts to conjugate a hydrophilic entity onto the intermediate product, allowing the formation of a more polar metabolite that can be excreted in the urine or bile [14]. Genetic variants of DME genes such as SNP, insertion/deletion, and gene duplication may alter either the expression level or the functional activity of an enzyme, resulting in aberrant pharmacokinetics and ultimately leading to ADR. With the substantial progress in current pharmacogenomic studies, numerous genetic variants of DME have been identified as predisposing factors to ADRs. A comprehensive review that covers the genetic variations in phase I and phase II DMEs to the safety and toxicity of drug therapy has been published recently [15]. Here, we summarize only “known valid” DME biomarkers and their effects on drug safety.

The majority of DMEs belong to the CYP gene superfamily, which encodes a phase I enzyme family, the cytochrome p450 superfamily [16]. Polymorphisms in CYP1A2, CYP2C9, CYP2C19, and CYP2D6 have been evaluated to contribute to clinically significant differences in exposure to several drugs [17]. Among these SNPs, several CYP2C9 variants (predominantly CYP2C9∗2 and ∗3 alleles) are relevant to adverse effects of numerous antiepileptics, antidepressants, nonsteroidal anti-inflammatory agents, sulfonylurea antidiabetic drugs and, most critically, oral anticoagulants (e.g., acenocoumarol and warfarin) [18]. Myriads of clinical studies have shown that the CYP2C9 polymorphism should be considered in warfarin therapy [19]. Similar information is known for another member of CYP2C subfamily, CYP2C19. The CYP2C19∗2 allele was associated with a marked decrease in platelet responsiveness to clopidogrel, an anticoagulant [20] while the pharmacokinetics of citalopram, an antidepressant, were influenced by the CYP2C19∗2 and CYP2C19∗17 alleles [21, 22]. Dose adjustments for these drugs based on CYP2C19 genotypes have been suggested. In addition, CYP2D6, another most extensively studied polymorphic, CYP, is involved in the metabolism of a large number of drugs, such as antiarrhythmics, tricyclic and second-generation antidepressants, antipsychotics, *β*-blockers, opioid analgesics, and anticancer drugs [23]. Carriers of duplicated variants of CYP2D6 (CYP2D6∗2) have been shown to be susceptible to the ADR of codeine treatment [24, 25]. Cumulative pharmacokinetic data from patients and healthy volunteers have also suggested a reduction in drug dosage for several antidepressants based on CYP2D6 phenotypes [25].

Furthermore, genetic polymorphisms of phase II DMEs are also known to influence the drug metabolism and the development of ADRs. An association of the genetic variation in the promoter region of uridine diphosphate glucuronosyltransferase 1A1 gene (UGT1A1∗28) with irinotecan-associated toxicity has been prescribed [26, 27]. The wild-type allele of UGT1A1 has six TA repeats in the promoter region while the UGT1A1∗28 has seven TA repeats, producing an enzyme with reduced activity [26]. Another notable example is the involvement of thiopurine S-methyltransferase (TPMT) allelic variants (predominantly TPMT∗2, TPMT∗3A, and TPMT∗3C) in mercaptopurine- or azathioprine-related adverse events [28–30]. Other phase II DMEs whose genetic polymorphisms have been correlated with drug toxicity are* N*-acetyltransferase type I (NAT1) and type II (NAT2) [31]. By comparison with the NAT2 genes, only a small number of NAT1 variants result in alteration of phenotypes. An increased incidence of drug toxicities in subjects carrying polymorphic NAT2 alleles has been reported when received hydralazine and sulfasalazine [32–34].

## 4. Drug-Transporter Proteins

Drug-transporter proteins (DTPs) represent another group of important determinants that govern the pharmacokinetics. These transporters are integral membrane proteins that mediate the influx or efflux transport of drug metabolites across the membrane using active and passive mechanisms [35]. Influx DTPs are mainly composed of the solute carrier (SLC) superfamily, including the organic cation transporters (OCTs), the multidrug and toxin extrusion (MATE) transporters, the organic anion transporters (OATs), and the organic anion transporting polypeptides (OATPs), while efflux transporters consist of members of the ATP-binding cassette (ABC) superfamily, such as P-glycoprotein (P-gp/MDR1), breast cancer resistance protein (BCRP), and transporters of the multidrug resistance-associated protein (MRP) family. For a more detailed description regarding the impact of DTPs on drug efficacy and toxicity, refer to a recent comprehensive review [36]. Here, we highlight those with well-defined pharmacogenomic roles in the development of ADRs.

OATP1B1, encoded by SLCO1B1, remains one of the most extensively studied influx DTPs, owing to the prevalence of clinically relevant polymorphisms [37]. A well-characterized SLCO1B1 variant is the loss-of-function polymorphism c.521T>C (rs4149056). The genetic association of rs4149056 with myopathy induced by simvastatin, a 3-hydroxy-3-methylglutaryl-coenzyme (HMG-CoA) reductase inhibitor used for controlling elevated cholesterol, has been identified [38, 39]. It is, thus, recommended that genetic tests of SLCO1B1 genotypes may be clinically useful tools for preventing simvastatin-induced muscle toxicity [40]. Similar finding was also observed in the OCTs, whose expressions and activities are crucial for the delivery of antineoplastics to the target tissues. A SNP of OCT2 gene (SLC22A2), rs316019, was found to be associated with reduced nephrotoxicity from cisplatin in cancer patients [41]. This observation was supported by the pharmacokinetic study of cisplatin in OCT2 knockout mice. In addition, another group of influx DTPs that moves small organic anions against their concentration gradient using a Na+ gradient is the OAT family. Of particular significance in drug disposition are OAT1 and OAT3, encoded by SLC22A6 and SLC22A8, respectively. A SNP in the intergenic region between SLC22A6 and SLC22A8 (rs10792367) was recently identified to be associated with hypertension to hydrochlorothiazide [42], although association studies of genetic variants in genes encoding OATs with changes in drug disposition are very limited.

Polymorphisms in efflux transporters are also known to be involved in the toxicity to drug treatment or predisposition to ADRs. A noteworthy example is the pharmacogenomic finding regarding P-glycoprotein (ABCB1/MDR1), the first human ABC transporter gene formerly characterized through its ability to confer a multidrug resistant (MDR) phenotype to certain chemotherapy drugs in cancer cells [43]. Among numerous variants of ABCB1 identified, a correlation of the ABCB1 3435T>C (rs1045642) was observed with cyclosporine-induced nephrotoxicity [44, 45]. In addition, functional effects of genetic variants in the ABCB1 gene have been considered as haplotypes rather than independent SNPs, as the use of ABCB1 haplotypes has been applied to predict the pharmacokinetics of many drugs [46–48]. Other lines of evidence also indicate that the SNPs of another ABC gene, ABCC4 (encoding MRP4), showed an association with ADRs induced by cyclophosphamide and methotrexate in cancer patients [49, 50]. A brief summary of the association between genetic variations involved in pharmacokinetics and pharmacodynamics and their related ADRs is shown in Table 1.

## 5. Human Leukocyte Antigen (HLA)

Other than genes involved in pharmacokinetics and pharmacodynamics, an immune etiology has been suggested for a great number of ADRs, in particular, type B reactions [76]. Many attempts to search for the associations with specific HLAs have been made, and the findings often are drug and ethnicity specific as summarized in Table 2. Such type of ADRs is recognized as drug-induced hypersensitivity reactions that involves major histocompatibility- (MHC-) restricted drug presentation and subsequent activation of specific immune responses. Two types of the classical MHC molecules mediate this process: the MHC class I molecules, expressed by most nucleated cells, and the MHC class II molecules, expressed by specialized antigen-presenting cells (APCs). In humans, the classical MHC class I molecule is encoded by three loci known as HLA-A, HLA-B, and HLA-C; the classical MHC class II molecule is encoded by three loci known as HLA-DR, HLA-DQ, and HLA-DP. MHC class I and class II molecules may regulate the drug hypersensitivity by presenting antigenic drugs to CD8+ (cytotoxic) and CD4+ (helper or regulatory) T cells, respectively. Because drugs are usually too small to likely trigger an immunogenic response, several mechanistic models, including the hapten/prohapten model, the p-i model, and the altered repertoire model, have been proposed to explain how small molecular synthetic compounds are recognized by T cells in an MHC-dependent/independent fashion. The hapten/prohapten concept proposes that the drug or its metabolite (hapten/prohapten) reacts with a self-protein through covalent binding to generate a haptenated,* de novo* product. This product then undergoes antigen processing to create a novel MHC ligand that is loaded onto the MHC and trafficked to the cell surface, where it activates antigen-specific T lymphocytes [77, 78]. In addition, a second concept, the p-i (pharmacological interaction with immune receptors) model, describes that a noncovalent, labile interaction of the drug with the MHC receptor at the cell surface is involved in MHC-dependent/independent T-cell stimulation by various drugs [79]. Neither cellular metabolism nor antigen processing is required in such an interaction. This model, to some extent, explains certain cases where drug hypersensitivity occurs rapidly, since the immunogenic complexes produced by drug presentation are unlikely to depend on antigen processing and cellular metabolism. Another concept, the altered repertoire model, has recently been proposed, according to which the drugs or its metabolites can bind noncovalently within the pocket of the peptide binding groove of certain MHC molecules with extraordinary specificity, allowing a new repertoire of endogenous self-peptides to be bound and presented. This concept is supported by the findings from various studies of abacavir-mediated drug hypersensitivity that the binding of abacavir to the antigen-binding cleft of HLA-B∗5701 sterically hindered the binding of the original repertoire of peptides, thereby prompting the binding of a new repertoire of peptides bearing immunogenic neoepitopes [80–82].

The striking examples of HLA associations with ADRs are HLA-B∗1502 with carbamazepine-induced Stevens-Johnson syndrome (SJS) and toxic epidermal necrolysis (TEN) in many regions of Southeast Asia [59–61], HLA-B∗5801 with allopurinol-induced SJS/TEN/hypersensitivity syndrome (HSS) [53–55], and HLA-B∗5701 with abacavir-induced hypersensitivity syndrome in the Caucasian population [51, 52]. These HLA-linked ADRs typically occur in defined populations owing to the prevalence of the specific alleles. The knowledge gained from such pharmacogenomic studies has led to a further development of genetic tests for identifying individuals at risk of these serious conditions [76]. Moreover, regardless of the diversity of genetic backgrounds and the difference in sample sizes examined, other HLA-drug associations that contribute to the pathogenesis of ADRs have been reported: HLA-A∗3101 and HLA-B∗1511 with carbamazepine-induced HSS [63, 65, 66, 83], HLA-B∗1301 with dapsone-induced hypersensitivity syndrome [68], HLA-B∗1502 with phenytoin-induced SJS/TEN [59, 70], HLA-B∗3505 and HLA-BRB1∗0101 with nevirapine-induced cutaneous ADRs [74, 84], HLA-B∗5701 with flucloxacillin-induced hepatitis [69], HLA-DPB1∗0301 with aspirin-induced asthma [85], and HLA-DQA1∗0201 with lapatinib-induced hepatotoxicity [86].

## 6. Miscellaneous

In addition to those mentioned above, genetic variations of many genes that are unrelated to pharmacokinetics/pharmacodynamics and HLA-restricted immune responses have been found to be associated with drug toxicity. These include, but not limited to, various cytokine gene promoters [87–89], epidermal growth factor receptor (EGFR) [90, 91], Fc gamma receptor [92], and microRNAs [93].

## 7. Conclusions

A decade has passed since the completion of the human genome project. During this period, human genetic research has revealed that the genetic backgrounds between the individuals can contribute to differences in the susceptibility to various ADRs. Thousands of genetic variations that are associated with drug safety and toxicity have been identified, many of which have shown high accuracy at predicting drug responses and adverse events. However, the molecular mechanisms through which these biomarkers influence disease risk and/or phenotypic expression still need to be further elucidated. More importantly, to determine which patients will benefit or suffer from a particular drug, the major challenge lies in translating the findings into clinical practice, which perceivably is a key component of the advancement to “personalized medicine.”

## Figures and Tables

**Table 1:** Associations between genetic variants involved in pharmacokinetics and pharmacodynamics and their related ADRs.

Genetic variants	ADR	Drug	Reference
ABCB1 (rs1045642)	Nephrotoxicity	Cyclosporine	[44, 45]
ABCC4 (rs9561778)	Leukopenia/toxicity	Cyclophosphamide	[49]
CYP2C19∗2	Decreased platelet responsiveness	Clopidogrel	[20]
CYP2C19∗2, CYP2C19∗17	Altered pharmacokinetics	Citalopram	[21, 22]
CYP2D6∗2	Opioid intoxication	Codeine	[24]
Polymorphic NAT2	Toxicity	Hydralazine, sulfasalazine	[32–34]
SLC22A2 (rs316019)	Reduced nephrotoxicity	Cisplatin	[41]
SLCO1B1 (rs4149056)	Myopathy	Simvastatin	[38, 39]
TPMT∗2, TPMT∗3A, TPMT∗3C	Hematologic toxicity	Mercaptopurine, azathioprine	[30]
UGT1A1∗28	Toxicity	Irinotecan	[26, 27]

ABCB1: ATP-binding cassette subfamily B member 1; ABCC4: ATP-binding cassette subfamily C member 4; CYP: cytochrome p450 superfamily; NAT2: *N*-acetyltransferase type II; SLC22A2: solute carrier family 22 member 2; SLCO1B1: solute carrier organic anion transporter family member 1B1; TPMT: thiopurine S-methyltransferase; UGT1A1: uridine diphosphate glucuronosyltransferase 1A1.

**Table 2:** Genetic associations of HLA alleles with severe ADRs.

Drug	HLA allele	ADR	Ethnic population	Reference
Abacavir	B∗5701	HSS	Caucasian	[51, 52]

Allopurinol	B∗5801	SJS/TEN/HSS	Han Chinese, Thai, Japanese,	[53–56]
			European	

Aminopenicillins	A∗2, DRw52	DHS	Italian	[57]

Amoxicillin-clavulanate	A∗0201	DILI	Caucasian	[58]
	DQB1∗0602			

Carbamazepine	B∗1502	SJS/TEN	Han Chinese, Thai, Indian	[59–62]
	B∗1511		Japanese	[63]
	B∗5901		Japanese	[64]
	A∗3101	HSS	Han Chinese, Japanese,	[65–67]
			European	

Dapsone	B∗1301	HSS	Han Chinese	[68]

Flucloxacillin	B∗5701	DILI	Caucasian	[69]

Lamotrigine	B∗1502, B∗38	SJS/TEN	Han Chinese	[55, 70, 71]
	B∗5801, A∗6801,		European	
	Cw∗0718,			
	DQB1∗0609,			
	DRB1∗1301			

Lumiracoxib	DRB1∗1501	DILI	Multiple populations	[72]
	DQB1∗0602			
	DRB5∗0101			
	DQA1∗0102			

Methazolamide	B∗5901, CW∗0102	SJS/TEN	Korean, Japanese	[73]

Nevirapine	B∗3505	DHS	Thai	[74]
	DRB1*∗*0101	DHS	Australian	[75]

Oxicam	B∗73, A∗2, B∗12	SJS/TEN	European	[55, 71]

Oxcarbazepine	B∗1502	SJS/TEN	Han Chinese	[70]

Phenytoin	B∗1502	SJS/TEN	Han Chinese, Thai	[59, 70]

Sulfamethoxazole	B∗38	SJS/TEN	European	[55]

HLA: human leukocyte antigen; HSS: hypersensitivity syndrome; SJS/TEN: Stevens-Johnson syndrome/toxic epidermal necrolysis; DHS: delayed-type hypersensitivity reaction; DILI: drug-induced liver injury.
